# Mental Health Crisis and Policing Demand

**DOI:** 10.23889/ijpds.v9i5.2506

**Published:** 2024-09-18

**Authors:** Ian Sobotka, Lauren Cipriano, Doug Coyle, Deva Thiruchelvam, Baiju Shah

**Affiliations:** 1 Ivey School of Business; 2 University of Ottawa; 3 ICES; 4 University of Toronto; 5 Sunnybrook Health Sciences Centre

## Objective

To develop a microsimulation model for type 2 diabetes using population-level real-world data. Such a model allows for the synthesis of multiple data sources for comparative effectiveness analysis related to a variety of correlated outcomes.

## Approach

The model was built using health state features including sex, age, diabetes duration, laboratory test results, and a history of major acute events. Features update using a cycle length of one month. Events modelled include myocardial infarction, stroke, heart failure, amputation and death. Model outputs include counts of events, lifetime healthcare costs and quality-adjusted life-years (QALYs). We then used the model to calculate the number of events that could be averted if a population with type 2 diabetes achieved treatment targets for HbA1c, blood pressure and LDL-cholesterol for 10 years.

## Results

Bringing all 60-year-olds with type 2 diabetes into target for 10 years would result in annual event reductions of 87.0 per 100,000 person-years for myocardial infarction, 49.4 for stroke, and 166.8 for heart failure. QALYs would improve by 1,155 per 100,000 patients. For 75-year-olds, annual event reductions would be 178.2, 56.8 and 261.6 per 100,000 person-years, respectively, and QALYs would improve by 2,121 per 100,000 patients.

## Conclusions

Population-level real-world data can be used to develop microsimulation models for type 2 diabetes that estimate long-term event risks, mortality and healthcare costs. This model is capable of comparative effectiveness and cost-effectiveness analysis of novel therapies in diabetes.

